# Forest Disturbance Monitoring Using Cloud-Based Sentinel-2 Satellite Imagery and Machine Learning

**DOI:** 10.3390/jimaging10010014

**Published:** 2024-01-05

**Authors:** Tamás Molnár, Géza Király

**Affiliations:** 1 Forest Research Institute, Department of Forest Ecology and Silviculture, University of Sopron, Bajcsy-Zsilinszky u 4, 9400 Sopron, Hungary; 2 Department of Surveying, Geoinformatics and Remote Sensing, University of Sopron, Bajcsy-Zsilinszky u 4, 9400 Sopron, Hungary; kiraly.geza@uni-sopron.hu

**Keywords:** forest damage monitoring, Sentinel-2 satellite imagery, vegetation index, random forest

## Abstract

Forest damage has become more frequent in Hungary in the last decades, and remote sensing offers a powerful tool for monitoring them rapidly and cost-effectively. A combined approach was developed to utilise high-resolution ESA Sentinel-2 satellite imagery and Google Earth Engine cloud computing and field-based forest inventory data. Maps and charts were derived from vegetation indices (NDVI and Z∙NDVI) of satellite images to detect forest disturbances in the Hungarian study site for the period of 2017–2020. The NDVI maps were classified to reveal forest disturbances, and the cloud-based method successfully showed drought and frost damage in the oak-dominated Nagyerdő forest of Debrecen. Differences in the reactions to damage between tree species were visible on the index maps; therefore, a random forest machine learning classifier was applied to show the spatial distribution of dominant species. An accuracy assessment was accomplished with confusion matrices that compared classified index maps to field-surveyed data, demonstrating 99.1% producer, 71% user, and 71% total accuracies for forest damage and 81.9% for tree species. Based on the results of this study and the resilience of Google Earth Engine, the presented method has the potential to be extended to monitor all of Hungary in a faster, more accurate way using systematically collected field-data, the latest satellite imagery, and artificial intelligence.

## 1. Introduction

The increasing occurrence of combined biotic and abiotic disturbances in recent years is putting forests at a greater risk of severe damage all around Europe [1]. The frequency and severity of large-scale droughts, fires, and insect outbreaks have reached unprecedented levels [2]. In addition, climate change exacerbates both types of disturbances [3]; thus, studying forest ecosystems on the European level is necessary and could reveal negative responses to forest disturbances.

As is the case in all of Europe, in Hungary, the occurrences of abiotic (windfall, snow break, droughts, flood, fire, etc.), and biotic (insects, wildlife, virus, and fungi) disturbances have increased in their number and intensity in recent decades, which is visible in the damage trends as well [4]. When tree stands are damaged, the yield significantly decreases and mortality increases. This results in a lower photosynthetic activity and tree vitality. Damage chains can appear as well, since the probability of biotic damage made by insects is higher after severe abiotic damage such as a drought, when the defence mechanisms of trees are weakened and more vulnerable [5]. Both biotic and abiotic disturbances and their combinations concern the scientific community, since the vulnerability of forests increases under climate change. Not only the declination of forest health but also massive tree mortality was reported, which has great ecological and economic importance [6].

Both biotic and abiotic damage can be detected using remote sensing, on which complex forest monitoring systems can be built [7,8]. Using remote sensing (RS), we can monitor the physical characteristics of forests from a distance by utilising emitted and reflected solar radiation [9]. Satellite-based RS methods offer great opportunities to collect comprehensive data about the current state of health and temporal dynamics of forests, with a high temporal (from 2 to 5 days up to intraday) and spatial (from 10 m to submeter) resolution [10]. In addition to abiotic and biotic damage, satellite images detect anthropogenic activities affecting forest health, such as forest management with clearcuts, selective cutting, and reforestation, which makes them ideal for large-area forest mapping and monitoring of all disturbance types [11].

The utilisation of optical satellite image series data provides a powerful, precise and cost-efficient means of detecting changes in forest health [12]. The European Space Agency’s (ESA) Sentinel-2 (S-2) satellite imagery, available since 2015, with a 10 × 10 m spatial resolution and 2–5-day revisit time, has multiplied the available amount of geospatial data, making it possible to develop forest monitoring approaches and systems, even with harmonised, long-term datasets.

Forest monitoring approaches can be divided into two groups: systems and services. Systems are created for entire countries, utilising a national database, developed by professionals from institutes with specific forestry knowledge. The results are published on interactive web pages, and the systems’ services are updated regularly. A forest monitoring system was developed in Hungary, called ‘TEMRE’, capable of detecting different types of damage based on vegetation indices derived from MODIS [13] and Sentinel-2 and -3 imageries [14]. The changes in forest health are monitored in wall-to-wall systems, utilising harmonised Landsat and Sentinel-2 image series in the Slovak Republic [15] and Sentinel-2 and Planet in the Czech Republic [16]. The Czech system focuses on spruce bark beetle (*Ips typhographus*) damage, which has become a severe issue lately in several European countries [17]. Buras et al. [2] observed and quantified drought impacts with the German forest condition monitor utilising MODIS images of forests in Germany and Europe, which is also a frequent problem all over the continent.

The second group of monitoring approaches is services. They are usually developed and tested for study areas and specific reasons, e.g., a natural disaster. The results are published in articles, and the maps are not available online for access. The scale of these services ranges from nationwide ones to smaller study areas.

Landsat satellites were utilised to investigate how the spatial and temporal characteristics of the Landsat archive can support forest monitoring in Finland [18]. Kern et al. [19] focused on the biotic damage in certain parts of Hungary and Croatia caused by the oak lace bug (*Corythucha arcuata*), while other Sentinel-based development projects have begun in several countries (Poland, Czech Republic, and Slovak Republic) for several reasons including forest health change [20,21], treefall gap [22], defoliation [23], and forest succession detection [24]. 

In both types of monitoring approaches, machine learning (ML) can be applied, which is an application of artificial intelligence that can be used in the Google Earth Engine (GEE) cloud computing platform [25] to expand the potential of monitoring forest health disturbances [26], identifying forest damage or classify tree species [27], which helps interpretation of forest maps more properly. ML algorithms like random forest (RF), minimum distance estimation, support vector machine, k-nearest neighbour regression, and gradient boost regression tree could be applied [28,29] to achieve monitoring goals. This study applied the RF algorithm, which is suitable for vegetation [30] and land cover monitoring [31], but also tree species classification [32]. The above-mentioned ML methods could potentially provide valuable information about forest health. Hence, they could be used to predict future states, which could further the enhance monitoring efficiency.

The main objective of this study was to test the GEE cloud computing method on Sentinel-2 imagery to reveal spatial and temporal changes in the forest cover and health in Hungary. The GEE is capable of executing the monitoring tasks in the online cloud: accessing, storing, processing, analysing, and visualising big data enables the fast and convenient monitoring of forests [33] and the effect of forest disturbances [34]. Cloud processing is a fast and powerful tool for monitoring [35], which is ideal for our goal.

The other objective was to test whether the validation of satellite maps is possible using ground-based datasets of the Hungarian Forestry Database (FD) and Hungarian National Forest Damage Registration System, where data are collected and stored regularly for the whole country in a uniform way. The ultimate goal is to automatise the monitoring process using both the remotely sensed and field-based datasets in a wall-to-wall monitoring system.

## 2. Materials and Methods

### 2.1. Study Site

**Figure 1:**
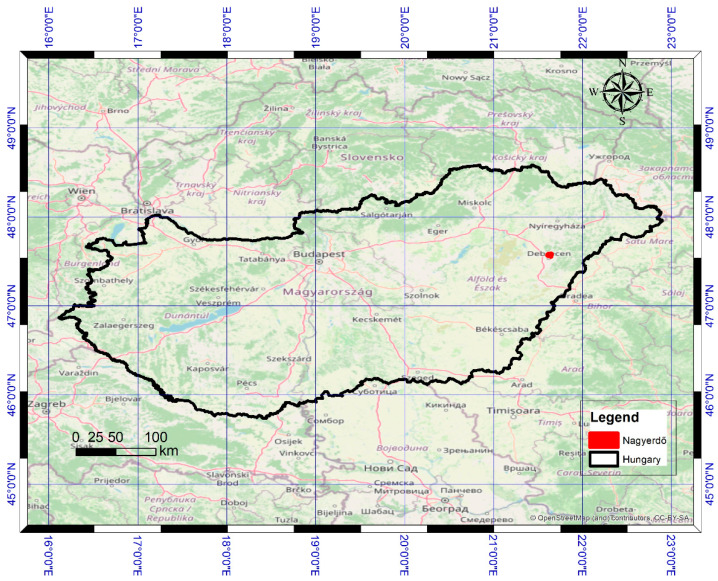
Location of the Nagyerdő in Eastern Hungary, north of the city of Debrecen.

The Nagyerdő (Great Forest) of Debrecen is situated north of the city of Debrecen (21.63° N, 47.57° E) in eastern Hungary (Figure 1) and covers a 1092 ha, contiguous, protected area [36]. In the past, the loess and sandy soils in the area had a favourable water supply that maintained large, continuous forests. Today, a drier climate, river regulations, and forest cutting have reduced the forest to smaller patches. 

The most typical forest communities in the Nagyerdő are oak with lily of the valley (*Convallario—Quercetum roboris*); oak–hornbeam (*Querco robori—Carpinetum*) and open oak forests on sand (*Festuco rupicolae—Quercetum roboris*), which were common in previous times, have disappeared. 

The tree composition of the Nagyerdő includes multiple species, but the oldest, largest, and most important, protected pedunculate oaks (*Quercus robur*) can be found in the old forest. Other domestic species like silver poplar (Populus alba), wild cherry (Prunus avium), Tatar maple (*Acer tataricum*), field maple (*Acer campestre*), field elm (*Ulmus minor*), wych elm (*Ulmus glabra*), large-leaved lime (*Tilia platyphyllos*), or silver lime (*Tilia tormentosa*) can be found too, while black locust (*Robinia pseudoacacia*), Scots pine (*Pinus sylvestris*), and red oak (*Quercus rubra*) are the most frequently found foreign species.

The forest has been suffering for decades from increasing abiotic damage as well as from more and more frequent drought stress during the spring and summer due to decreased precipitation and a lower groundwater level, resulting in a decreasing yield and lower vitality [37], while ice damage takes place during springs when seedlings and young trees freeze, and game damage is also observed in sites of reforestation. 

The forest was divided into four quarters; drought and frost damage occurred mostly in the NW and NE quarters, but it also occurred in the SW and SE quarters, to a lesser degree. A total of 131 forest compartments out of 394 (33%) were damaged.

### 2.2. Satellite-Based Dataset

Sentinel-2 imagery from the European Space Agency has been available since 2015, with a 2–5-day revisit time, high 10 × 10–60 × 60 m spatial resolution, and a free and open data policy, which makes it ideal for forest monitoring (ESA 2022). The multispectral instrument measures the radiance of the surface of Earth in 13 spectral bands (Table 1).

**Figure 2:**
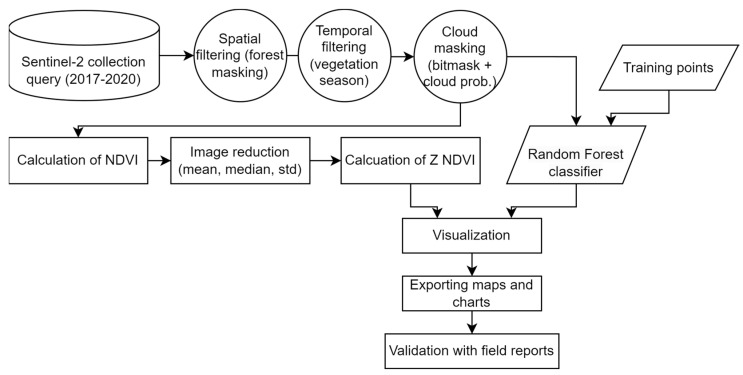
Methodological flowchart of processing, visualising, and exporting Sentinel-2 imagery in GEE for forest damage monitoring and tree species classification.

Sentinel-2 satellite data accessing, processing, analysis, and visualisation was performed online in Google Cloud using the application interface of GEE [38]. GEE uses both JavaScript and Python programming languages; here, JavaScript was used. The method used in GEE consists of several steps, shown in the flowchart (Figure 2). In order to create high-quality satellite composites for the study period, spatial, temporal, and quality filtering and masking were applied. Vegetation indices and classified forest maps were derived from these composites aiming to show the vegetation health state and species composition.

The Sentinel-2 L2A satellite imagery of the ESA provided the basis for the remote-sensing survey at a 10 × 10 m spatial resolution as the first step in the process. The bottom-of-atmosphere reflectance-based (level 2) annual composites were created with GEE for each year between 2017 and 2020 using spatial, temporal, and quality filtering for the region of interest (ROI), time window, and cloud coverage. The annual composites were made from the S-2_SR collection by applying the pixel-wise filters and reducers to create cloud-free images for vegetation periods. 

Spatial filtering using filterBounds was calibrated to filter pixels outside of the study area (or ROI), which was given in a shapefile polygon and a WGS84 (EPSG 4326) coordinate reference system. The polygons are from the Hungarian Forest Database, and they were filtered to exclude forest compartments with water surfaces, roads, buildings, openings, nursery gardens, and keep only forest-covered areas.

Similarly, we defined a time window using filterDate based on the vegetation period of each year (e.g., filterDate (‘14 April 2017’, ‘15 October 2017’)) to keep pixels from the active state of the vegetation. Temporal filtering is also important for reducing the dataset, since GEE is not capable of processing large-sized dataset for vast areas and longer periods. Altogether, 95 images were used for creating composites.

Cloud filtering was completed in two steps. The first step involved prefiltering cloudy pixels with the metadata of Sentinel-2 (ee.Filter.lte (‘CLOUDY_PIXEL_PERCENTAGE’, 5)) that were less than or equal to 5% of coverage, which was applied on the satellite image collection. The maximum 5% of cloud coverage limit was necessary to keep enough images for mosaicking. The second step utilised the QA10 bitmask, where 0 values of 10 and 11 bits refer to cloudless pixels. The two-step filtering resulted in a nearly cloud-free image collection.

The second step involved the calculation of vegetation indices, where the normalised difference vegetation index (NDVI) (1) and standardised Z∙NDVI (2) were calculated. 

The normalised difference vegetation index (NDVI) (1) is the most widely used index for indirectly measuring the photosynthetic activity of vegetation. It is calculated according to the following formula [39]:

NDVI = (NIR − RED)/(NIR + RED) (1)

where RED is band 4 and NIR (near infrared) is band 8. The high NDVI values mean healthy vegetation with strong photosynthetic activity, while low values refer to unhealthy vegetation or lower vegetation cover. The ee.image.normalizedDifference ([4,8]) function was used to calculate the NDVI in GEE.

To exhibit greater interannual and spatial changes and show vegetation anomalies more efficiently, standardised NDVI values (Z·NDVI) (2) [40] were calculated from the original NDVI:

Z·NDVI = (NDVI − NDVI_mean_)/NDVI_std_ (2)

where NDVI is the NDVI of the actual year, NDVI_mean_ is the multiple-year average of NDVIs (2017–2020 here), and NDVI_std_ is the standard deviation of NDVI values (2017–2020). Using a long-term mean and standard deviation helps to show actual change unlike the NDVI does, since it shows an actual state in itself.

The third step was the aggregation of the dataset of VIs with reducers, which is needed to visualise and export the dataset while not exceed the computing limitations of GEE. Different filters, such as median (.median()), mean (.mean()), and standard deviation (STD) (.stdDev()), were used for reduction. The mean and STD are used in the formula of the Z∙NDVI, while the median is for displaying and exporting maps. The creation of true colour composites (RGB) was the fourth step, using red (B4), green (B3), and blue bands (B2), and a median filter. Without these reducers, GEE is not capable of calculating mosaics due to the limitations.

Setting the display parameters and properties was the next step: the map centre (with ROI polygon as the centre and zoom level), colour palettes (green–red transitional scale for NDVI), and borders (colour and width) were described for visualisation. These parameters were used in the sixth step, when the actual visualisation occurred on the GEE platform: RGB and VI maps and charts. The charts were made with the ui.Chart.image.series function over the complete dataset, with a median reducer. 

The seventh step involved the export of the maps with Export.image.toDrive and charts with ui.Chart.image.series to Google Drive and a PC. Further offline analysis was conducted using QGIS 3.22.3. open-source desktop GIS software (QGIS 3.22.3) [41].

VI maps were derived from S-2 composites for each year. The state index maps provided information on the condition of the forests in each year. A standardised NDVI was calculated using the time series of 2017–2020, presenting change with a deviation from median of the four years. 

The tree species classification was achieved with ee.Classifier.smileRandomForest classifier. A median composite of 2021 was used as the base of the dominant species classification: black locust, Scots pine, pedunculate oak, red oak, and clearcuts. All satellite bands were used in the classification as an input. The RF algorithm used 100 decision trees and 10 variables per split at 10 × 10 m spatial resolution.

Manually selected training points on the RGB image gave the base of the sampling; as training input, these points were utilised for the classification of tree species. Of the 123 points (15 black locust, 22 red oak, 27 Scots pine, 40 pedunculate oak, and 19 clearcut) selected using the geometry imports function, five classes were defined. The classes were based on the Hungarian Forestry Database’s ground-based tree species dataset. The RF-based classification was completed with the training data, and the classified map was exported using the image.toDrive function, which was later validated with ground-based data.

### 2.3. Ground-Based Dataset

Ground-based forest damage reports were applied for validation. The forest protection damage reports of the Hungarian National Forest Damage Registration System (HNFDRS) of the Hungarian National Land Centre are collected systematically since 2012, four times per year throughout the country on forest compartment levels.

**Figure 3:**
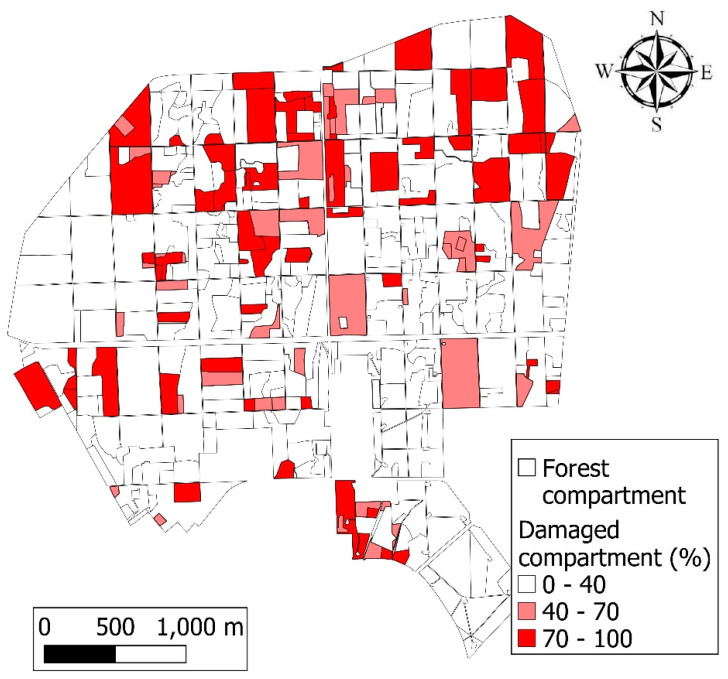
Field-based damage map of the Nagyerdő from 2017 to 2020. The most considerable severe damage was observed in the northern half, but 33% of the area of the forest was damaged overall.

In the damage reports, late frost was registered in young stands in 2017, while in 2018, 2019, and 2020 [42,43], drought, frost, and game damage took place in the Nagyerdő. The damage type is registered in the reports and is available on the HNFDRS website. The data contain information about damage frequency and intensity (or severity), which are given for each forest compartment expressed as the percentage of damaged trees for each tree species (0–100%) (Figure 3) and the intensity of damage (0–100%), respectively [43]. The damaged area was given in hectares from ground-based reports, while a new attribute, damage ratio (3), was calculated using the following formula, measured on a 0–100% scale:

Damage ratio = damaged area/total area × 100 (3)

Validation of the RS data with field data of forest health was achieved with confusion matrices. The damage registered in the forest damage reports was compared to Z∙NDVI values on the pixel level in QGIS 3.22.3 software. For this, the polygons of reports were rasterised to a 10 × 10 m resolution grid that is identical to the Sentinel-2 images in size. These rasters of field-based damage reports were reclassified according to the damage ratio: when it was below 30%, it was assigned a value of 0 (no damage), while above 30%, the value was 1 (damage). While regarding Z∙NDVI values, −0.5 was selected as a threshold. Every pixel was considered forest damage if the value was below the threshold and marked with 0, while all pixels exceeding the threshold were assigned a value of 1. The result maps were analysed using the accuracy assessment postprocessing function of the semi-automatic classification plugin (SCP) of QGIS in the form of confusion matrices [44].

To validate RF-based tree species classification results, the majority value was calculated from RF-based classified pixels in QGIS for each forest compartment with zonal statistics of the raster analysis function. The majority function showed the most frequent pixel value of the dominant tree species. The reclassified RF-based tree species map was compared to field data of the forestry database. The attribute of the dominant species of the forestry database was compared to the RF-based species. The forest compartment-level comparison was based on the number of agreements between field-based and classified tree species. In the MS Excel table, the if function was used to test logical agreement. If both classification and field data agreed, the operator said true; otherwise, it was false. The total accuracy was calculated from every forest compartment according to this formula (3):

Tree species total accuracy = correctly classified compartments/total compartments × 100 (4)

### 2.4. Statistical Analysis

We performed the Shapiro–Wilk test for normality on 100 randomly selected points from all Z∙NDVI maps. This method was applied by the authors of the Z∙NDVI formula [40], and 80% normality was achieved in that study. The points were created with random points in the layer bounds function of QGIS and analysed using Past 4.14 statistics software [45]. 

## 3. Results

Our study managed to show forest damage within the Nagyerdő of Debrecen. The field-based and remotely sensed datasets were suitable input for cloud computing, and machine learning was applied for damage detection and tree species classification. 

Normality tests were conducted on Z∙NDVI maps for each year and found that at *p* < 5%, their distribution was normal for at least 95% of the points in 2017, 2018, and 2019. In these years, *p* values were 0.37, 0.3, and 0.11, respectively, while in 2020, the *p* value was significantly lower (0.01), but it was not due to the outliers; the bins of Z∙NDVI values were distributed evenly.

**Figure 4:**
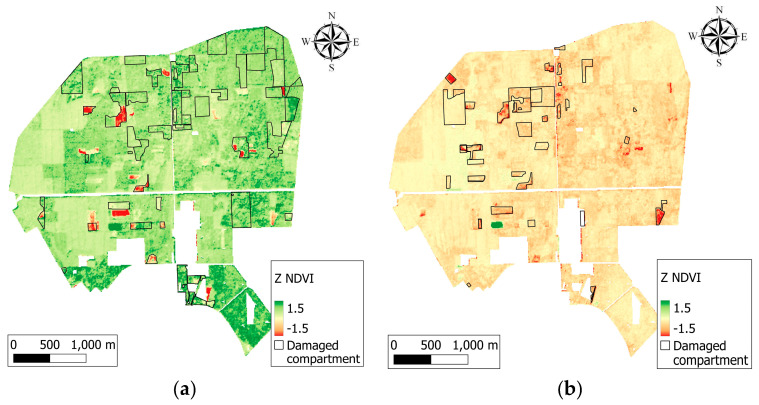

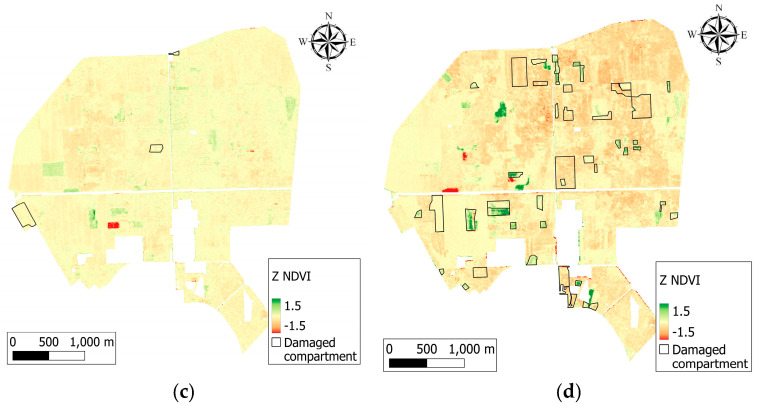
Annual Z·NDVI composites of the Nagyerdő in 2017 (**a**), 2018 (**b**), 2019 (**c**), and 2020 (**d**). In every year, clearcuts, droughts, and frost damage caused a significant drop of photosynthetic activity when each year was compared to the long-term mean. Regeneration was observed as well, and there were differences between tree species’ reactions to damage.

Forest health was successfully studied in the period of 2017–2020. The analysis of Z∙NDVI maps showed differences between the years (Figure 4). The map of the year 2017 contained large, healthy, dark green coloured areas with high Z∙NDVI values, but also some damaged, orange, or red areas with a rather open canopy or clearcuts. A decrement in Z∙NDVI values was detected in 2018 in the majority of the forest, with new areas of clearcuts. A positive anomaly appeared on the maps in 2019 and 2020 caused by artificial plantings after clearcuts and regeneration forests. However, generally positive changes were observed, although negative changes were also apparent in these years, like new clearcuts. Moreover, in 2020, the oak-dominated NE quarter of the forest became less vital. This negative change is due to drought stress to which pedunculate oak is sensitive [46].

Differences have been observed between the studied years. Both the RS-based and ground-based methods detected that several forest compartments were damaged in 2017. However, these differences are less evident in 2018. The year 2018 was generally drier than 2017, and the entire forest appeared to be in a less vital condition in accordance with the Z·NDVI values, while 2019 was a year with more positive values and fewer damage reports; thus, better condition. In 2020, the forest state declined again when more damage was reported and lower Z·NDVI values were observed, especially in the NE quarter, where a visible phenomenon shows the suffering of pedunculate oak-dominated stands, which lasts for decades due to drought stress caused by a lack of water and heat periods. However, there is no perfect match between the ground-based survey of disturbances and RS data, which could be due to weaker, no-disturbance changes in forests.

Pixel-wise comparisons of remotely sensed Z·NDVI values and ground-based damage reports were made using confusion matrices, calculated for each year, where the RS-based Z·NDVI was the classified, and the field reports were the reference values (Table 2). 

User’s (UA), producer’s (PA), and total accuracies (TA) were given for every year as well. The mean TA for all four years was 70.85% (Table 3). The PA was high (99–100%), but the UA varied between 62.7 and 74% and TA between 63.24 and 74.51%.

The differences between VI values within forest compartments are not exclusively due to damage but also to different tree species, which were observed on the RF-based classification and VI maps as well.

**Figure 5:**
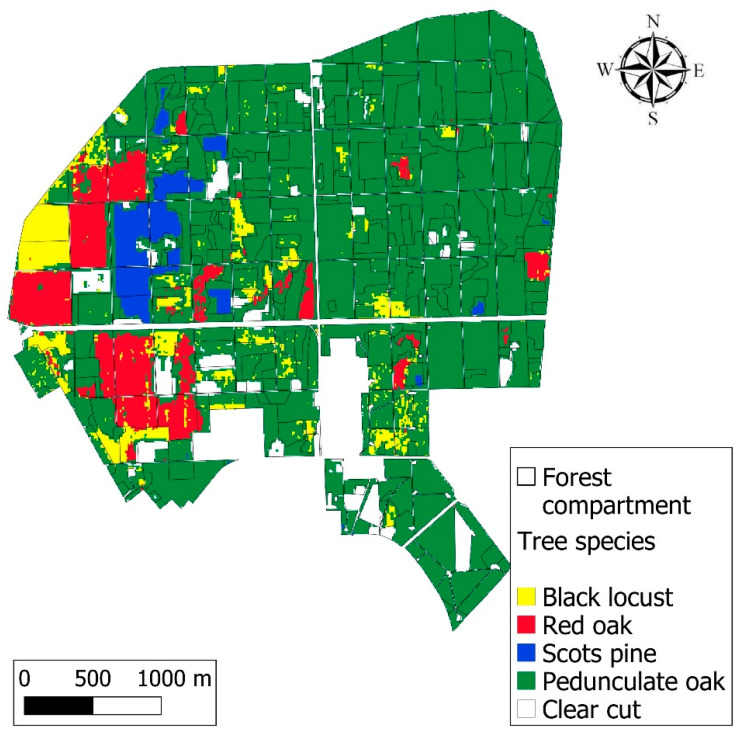
Dominant tree species of the Nagyerdő based on random forest classification of Sentinel-2 imagery from 2020.

RF-based classification showed that the NE and SE quarters were dominated homogeneously by pedunculate oak (Figure 5), with smaller patches of other species, while in the NW and SW quarters, the occurrences of black locust, red oak, and Scots pine were detected in coherent patches where species distribution often corresponded to forest compartment borders. 

The species comparison on a forest compartment level based on a majority attribute indicated a 76.1% accuracy for the five classes (four species plus clearcut). In 258 compartments of the total 339, there was an agreement on the species. However, we aimed to classify only the dominant species, while other compartments with secondary, tertiary, or quaternary mixed species resulted in a lower accuracy. Furthermore, there is an ongoing tree species change in several compartments when foreign species are exchanged with domestic pedunculate oak. That is the reason for compartment-sized clearcuts resulting in a lower classification accuracy. Only focusing on forest-covered compartments and excluding clearcuts, a higher total accuracy was registered: 81.79%.

The comparison of VIs and classified tree species maps indicated a connection between them, and domestic and foreign species reacted differently to forest damage which resulted in different VI scores. The varying VI values of different species were visible on the Z∙NDVI maps of 2017 and 2018 (Figure 4a,b), in the bottom part of the NW quarter, where black locust, red oak, Scots pine, and pedunculate oak-dominated stands were situated in a row. The stands with black locust and the pedunculate oak majority had lower vitality and VI values, while pine and red oak were in a better condition compared to them. Scots pine and pedunculate oak showed higher Z∙NDVI values in 2017 compared to the other species; however, in the following years, vitality oak stands decreased, and larger, less vital areas appeared in certain forest compartments in 2019 and 2020 due to drought stress; especially in the NE quarter (Figure 4c,d), the state of the pedunculate oaks continuously declined in the study period. Frost damage in young stands can be seen as well in 2017, 2018, and 2020 in several compartments in the NW and SW quarters. 

**Figure 6:**
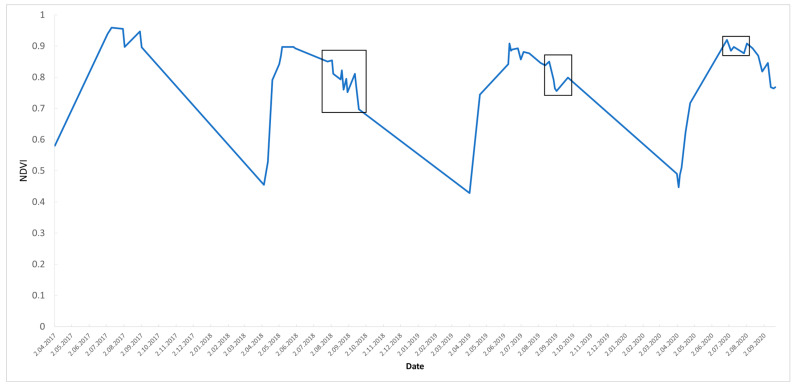
NDVI median graph of the Nagyerdő for the period 2017–2020. Deviations from the ideal curve are marked with a black frame, which could refer to forest damage.

In addition to maps, time series can also be analysed in charts; thus, these were created from the Sentinel-2 imagery showing a median NDVI curve of the Nagyerdő for the years 2017–2020 (Figure 6). A declination can be seen from the ideal curve in the vegetation period, when the curve ideally reaches 0.9 in the midsummer and gradually decreases in the fall. If the forest is damaged, it can be seen on the shape of the NDVI curve as well. Drought affected the NDVI curve in 2018, 2019, and 2020, and according to the severity of the damage, different drops were detected on the graph.

## 4. Discussion

The objective of our study was to utilise satellite imagery and field reports for forest damage monitoring and tree species classification conducted with Google Earth Engine and machine learning. The application of Sentinel-2 imagery in GEE has several advantages that made the forest monitoring successful; but it had also some limitations. The simultaneous utilisation of a combined remotely sensed and ground-based datasets system is possible in a wall-to-wall monitoring system, also in an automatised way; however, some known problems should be fixed before moving the system to an operational level.

Studies dealing with forest monitoring using GEE, Sentinel-2, or ML showed varied results from all over the world. Climate zones have an influence on the methods and results. A temperate forest degradation study was performed by Chen et al. [47] in Georgia using GEE and Landsat imagery, where the UA of the forest degradation class was 69% and the PA was 83%. In Germany, environmental drivers of drought in spruce stands were studied on NDVI curves derived from S-2 from 2016 to 2020, and they found that 38–45% of stands were damaged [48]. In Italy [49] and Croatia [50], forest disturbance events were mapped based on the difference between years (2017–2020 and 2016–2021, respectively). In these cases, combined biotic and abiotic factors were studied. In Portugal, the focus was on insect damage, which was identified with RF at an 81% accuracy [51]. 

To increase the accuracy of monitoring in a temperate climate, certain errors should be fixed, which we encountered while working with GEE. One problem was a high cloud cover which caused gaps in the dataset, especially in April, May, and June, months during which cloudless images of the area of interest were virtually unavailable for some years. The spring and early summer months (April, May, and June) are particularly important in the vegetation period because they mark the beginning of the growing season (leaf unfolding), with increasing photosynthetic activity. According to Swedish studies [52,53], these months provide valuable information concerning the spring phenological stage, and the RF classification is more accurate when these months are used. Still, cloud masking remains an existing problem in the S-2 dataset; however, an increasing number of refined datasets and algorithms such as the cloud probability dataset and the s2cloudless or Fmask algorithms, which are partly available in GEE [54]. The disadvantage of some algorithms like s2cloudless is the high computing capacity demand which easily causes timeout errors.

Tropical forest disturbances were studied by researchers since the largest, contiguous forests can be found there; these methods could be interesting in the European scaled plan. Chen et al. [55] used harmonised Landsat and Sentinel-2 data, showing 84.5% and 95.5% TAs for Tanzania and Brazil, respectively. A near real-time change detection approach with ML showed a 71–87% accuracy in Colombia and Mexico [56].

Later, in the boreal region, Yang [57] investigated biotic forest damage caused by the spruce bark beetle in Sweden on a multitemporal Sentinel-2 collection, with maximum likelihood and the enhanced vegetation index plus green normalised difference vegetation index, where an 89% TA was achieved. 

Another problem was the validation of the RS results with the ground-based dataset results. The large sampling area (several hectares) is a drawback of the ground-based dataset compared to high-resolution satellite pixels (10 × 10 m), which causes an uneven distribution of data. When scattered pixels are compared to entire forest compartments, the difference can be observed. In order to fix this problem, zonal basis median values of pixels were calculated for each compartment, which reduced the diversity of VI values and on one hand, made it harder to detect slight changes in the forest health state, but on the other hand, it made the datasets comparable. 

Our study also showed the advantage of the compartment-based comparison method’s easier application for practical use since field data from the forestry database are used by forest managers, and the VI maps were made for the same forest compartments and validated by the same datasets. It could reduce the workload of fieldwork; thus, using damage reports created by foresters could represent an alternative to the repetitive fieldwork triggered by each new research study. 

The date from the ground-based registration could also be problematic, since if field data are collected and reported after satellite image acquisition, there will not be overlap regarding damage in the same year. RS could indicate damage earlier than the ground survey, which is a useful feature to survey forest damage before going out to complete fieldwork. However, in some cases, the effects of dry periods appear in the following year instead of the studied year. The difference in sampling size is visible between the producer’s and user’s accuracies, where ~99% and 70% showed a notable difference in the accuracy of damage detection.

Machine learning was successfully applied to Sentinel-2 data aiming to classify tree species, supporting the interpretation of NDVI maps. The combination of the cloud systems of GEE and ML enabled the visualisation of the distribution of different forest types and different forest damage types in all the investigated years. However, some previous studies reported better results using multitemporal S-2 images with pedunculate oak, Scots pine, silver birch, dunkeld larch, and Norway spruce in Sweden, with RF resulting in an 88.2% total accuracy [52]. Fourteen S-2 images were used in the multitemporal imagery, resulting in an 88.2% TA, but for a single image in July, it was 75.9%, which is similar to our result. Also in Sweden, an 87% TA was obtained for the same species [53] with the Bayesian inference method, using 23 pieces of S-2 images. From Latvia, a 92–94% TA was reported with three S-2 images on Scots pine, Norway spruce, silver birch, and black alder [58]. With the RF method, Puletti et al. [32] obtained an 86.2% TA on four mixed forest types, emphasising that the multitemporal imagery made of different phenological periods was more accurate and proved to be better than single satellite images. However, the drawback of the presented work is that many studies focus on coniferous species, which are more easily classified than deciduous ones. Another reported issue was the accuracy of the deforestation class due to being small compared to other classes, so it is harder to detect [47].

It would be beneficial to test other ML methods on our study site as well, and several supervised and unsupervised algorithms are available in GEE besides RF. These algorithms can replace the process of downloading and processing satellite images as well as the need for external programs, since the whole process can be created in the cloud. 

On the other hand, ground-based datasets like forest damage reports cannot be uploaded directly to public GEE servers due to security issues and policies. This could make it impossible to carry out a fully online analysis, which would be desirable, especially on a larger scale (i.e., for the whole of Hungary or even Europe). Thus, the method has a potential for large-scale usage; however, its performance in different climatic regions needs to be validated first, and data security has to be taken into consideration. The ML can be used to find forest damage as well, in addition to the tree species classification, which could be an expansion of this study in the future. 

Utilised datasets could be expanded as well. Since drought has become a very serious problem both in Hungary and worldwide, climatic and meteorological datasets (precipitation, temperature, soil moisture, etc.) can be used to describe dry periods, as Birinyi et al. suggested, comparing corn yield to the NDVI and EVI in Hungary with GEE to demonstrate the correspondence of drought with VIs [59].

## 5. Conclusions

Based on our results, it can be said that the combination of satellite imagery and field-based reports could provide appropriate input for forest damage monitoring in GEE. We successfully identified different types of forest damage on Z∙NDVI maps in the surveyed four years (2017–2020), while tree species classification with RF was also successful. Both drought and frost damage were shown by the combination of RS-based and field datasets. This GEE and RF-based method is of great importance when forest damage is more and more frequent all around Europe due to climate change and the fact that several tree species are unable to adapt to warmer climatic conditions; thus, constant and accurate monitoring is needed to obtain data about forest health change.

The great advantage of GEE is resilience and flexibility: study sites can be modified easily, and the creation of maps and charts can be achieved rapidly for other areas covered by the same RS dataset, and Sentinel and Landsat satellites cover the mainland surfaces of Earth in 5–8 days, and their harmonised dataset (HSL) is available partly in GEE. 

We believe that greater cloud filtering is an essential part of forest monitoring to create denser time series of satellite imagery. However, the cloudy pixel percentage filtering and QA 10 bitmasking utilised in this study proved satisfactory results, since high-quality mosaics were made for each studied period, but there were gaps in the dataset during cloudy springs or summers. Other cloud-filtering algorithms like Fmask or s2cloudless can be tested on S-2 imagery and integrated into the GEE code, and with adequate cloudless images created with these new methods, the annual composites can be replaced with monthly or weekly ones. On these denser series, the seasonality in vegetation indices can also be studied more accurately. Although there could still be areas (high mountains and islands) with frequent cloud cover where it is not possible to achieve cloudless images for some periods, on mainland areas, the harmonisation of Sentinel and Landsat satellites could solve the problem of cloud coverage [60].

The great advantage of the RS-based method conducted using GEE is that it could support the forestry labour force with swift and accurate forest damage surveying in contrast with time-consuming, traditional fieldwork methods. The field reports and other field-based forestry data of national databases could support the expansion of the combined method, since they are produced for entire countries and no extra field measurements would be needed. That is one of the most important reasons why we used these databases in the study instead of conducting our own field measurements. As we hypothesised, the field reports can be used for validation despite the different scales; however, the accuracy can be improved. The results of our study can also help to carry out field measurements and reports more effectively. ML could also help determine the damage threshold instead of a manual selection and also detect trends in the time series of forest states, which sit outside these patterns, and identify the thresholds. 

To test our approach on a larger scale, the expansion to a wall-to-wall monitoring system would be desirable: first for Hungary and after, for Europe. Since in Europe, similar conditions and forest damage types can be found, the monitoring could be performed in a uniform way including data collection, processing, analysis, and visualisation as well. Cloud computing and machine learning have great significance in this method, since the whole monitoring process could run in the virtual cloud.

Besides spatial expansion, other types of forest damage could be surveyed with the GEE-based method, namely the spruce bark beetle and oak lace bug gradations, which have both caused massive damage in several European countries. This monitoring on the European level could show the outbreak and spread of the species from one country to another. The oak lace bug was studied in Hungary and Croatia in these terms but on MODIS imagery, not on Sentinel-2. The bark beetle-related studies also focused on study areas or single countries; thus, it would be interesting to try the method for a larger-scale damage survey using water indices, like NDWI.

Ultimately a wall-to-wall system could be created for the entirety of Hungary based on cloud-free Sentinel-2 composites, powered by GEE and supported by ML. The application of ML could ensure both a more accurate damage threshold determination and damage detection as well. A fully automatised, regularly updated, country-wide map would be a great asset for both researchers and practical foresters in forest surveying and damage monitoring. It could be part of the new EU Framework for Forest Monitoring and Strategic Plans as well as helping to develop an EU-wide forest observation framework.

## Figures and Tables

**Table 1:** Spectral bands of Sentinel-2 multispectral instrument.

Band Number	Bands	Central Wavelength (µm)	Resolution (m)
Band 1	Coastal aerosol	0.443	60
Band 2	Blue	0.490	10
Band 3	Green	0.560	10
Band 4	Red	0.665	10
Band 5	Vegetation red edge	0.705	20
Band 6	Vegetation red edge	0.740	20
Band 7	Vegetation red edge	0.783	20
Band 8	Near-infrared	0.842	10
Band 8A	Vegetation red edge	0.865	20
Band 9	Water vapour	0.945	60
Band 10	Short-wave infrared cirrus	1.375	60
Band 11	Short-wave infrared	1.610	20
Band 12	Short-wave infrared	2.190	20

**Table 2:** Confusion matrix of forest damage in the period of 2017–2020. Values are given as ratio (%).

		**2017**		**2018**	
		Reference			
		Damaged	Non-damaged	Damaged	Non-damaged
Classified	Damaged	72	25	55	32
	Non-damaged	3	0	1	12
		**2019**		**2020**	
		Reference			
		Damaged	Non-damaged	Damaged	Non-damaged
Classified	Damaged	75	25	72	28
	Non-damaged	0	0	0	0

**Table 3:** Accuracy assessment of forest damage datasets for the years 2017–2020.

	2017	2018	2019	2020	Mean
Producer’s accuracy (%)	99.19	99.89	100	99.42	99.63
User’s accuracy (%)	74.01	62.69	74.62	72.25	70.89
Total accuracy (%)	73.70	63.24	74.51	71.95	70.85

## Data Availability

The data presented in this study are available on request from the corresponding author.
